# Ortholog-based protein-protein interaction prediction and its application to inter-species interactions

**DOI:** 10.1186/1471-2105-9-S12-S11

**Published:** 2008-12-12

**Authors:** Sheng-An Lee, Cheng-hsiung Chan, Chi-Hung Tsai, Jin-Mei Lai, Feng-Sheng Wang, Cheng-Yan Kao, Chi-Ying F Huang

**Affiliations:** 1 Institute of Clinical Medicine, National Yang-Ming University, Taipei 112, Taiwan; 2 Institute of Bio-Pharmaceutical Sciences, National Yang-Ming University, Taipei 112, Taiwan; 3 Institute of Biotechnology in Medicine, National Yang-Ming University, Taipei 112, Taiwan; 4 Department of Computer Science and Information Engineering, National Taiwan University, Taipei 10617, Taiwan; 5 Institute for Information Industry, Taipei, Taiwan; 6 Department of Life Science, Fu-Jen Catholic University, Taipei Hsien 242, Taiwan; 7 Department of Chemical Engineering, National Chung Cheng University, Chia-Yi 621, Taiwan

## Abstract

**Background:**

The rapid growth of protein-protein interaction (PPI) data has led to the emergence of PPI network analysis. Despite advances in high-throughput techniques, the interactomes of several model organisms are still far from complete. Therefore, it is desirable to expand these interactomes with ortholog-based and other methods.

**Results:**

Orthologous pairs of 18 eukaryotic species were expanded and merged with experimental PPI datasets. The contributions of interologs from each species were evaluated. The expanded orthologous pairs enable the inference of interologs for various species. For example, more than 32,000 human interactions can be predicted. The same dataset has also been applied to the prediction of host-pathogen interactions. PPIs between *P. falciparum *calmodulin and several *H. sapiens *proteins are predicted, and these interactions may contribute to the maintenance of host cell Ca^2+ ^concentration. Using comparisons with Bayesian and structure-based approaches, interactions between putative HSP40 homologs of *P. falciparum *and the *H. sapiens *TNF receptor associated factor family are revealed, suggesting a role for these interactions in the interference of the human immune response to *P. falciparum*.

**Conclusion:**

The PPI datasets are available from POINT  and POINeT . Further development of methods to predict host-pathogen interactions should incorporate multiple approaches in order to improve sensitivity, and should facilitate the identification of targets for drug discovery and design.

## Background

Many genome-wide high throughput yeast two-hybrid analyses have generated PPI datasets for various model organisms. Moreover, systematic manual curation of human protein interactomes, including BioGRID [1], MIPS [2], IntAct [3], PINdb [4], DIP [5], HPRD [6] and MINT [7], has also generated significant, but far from complete, datasets. Therefore, in addition to an empirical screening of the interacting proteins of a given target, a comparative strategy should further facilitate functional annotation of uncharacterized proteins.

Using our knowledge of conserved interactions in other organisms (or interologs) [8] to elucidate the interacting networks of a particular target protein, we have previously established a publicly accessible and functional database, POINT (the Prediction Of INTeractome database) [9]. The application of a similar concept and the addition of further filtering criteria have recently been reported and, as a result, have produced many outstanding studies such as Ulysses [10], OPHID [11] and HomoMINT [12]. Recently, additional high-throughput yeast two-hybrid experiments have generated an enormous number of human PPIs [13,14], which now require assessments of their accuracy [15] and further evaluations using the concept of interologs. Conversely, interologs may be used to estimate the reliability of high throughput observations.

It is expected that the interactions between conserved orthologs, which are conserved genes and gene products in different species, will be conserved as well. However, accurate human interolog predictions inferred from different species are much less abundant than expected [6,12]. Additionally, some argue that interologs are less conserved than orthologs [12]. The extent to which ortholog-based PPI predictions can be applied has not been extensively analyzed.

In this work, orthologous pairs from 18 eukaryotic species have been expanded. Using experimental PPIs, interologs for these 18 species can be predicted and analyzed. This concept has been applied to host-pathogen PPI predictions. An analysis of predicted *H. sapiens*-*P. falciparum *interactions revealed PPIs that are highly related to the maintenance of Ca^2+ ^levels in host cells. When comparing this method to other prediction methods, we find that this approach can complement Bayesian statistical methods [16] and structure-based methods [17].

## Results and discussion

### Orthologs shared by *H. sapiens *and other model organisms

The complete ortholog matrix from 18 eukaryotic species is shown in Additional File 1: Table S1. For brevity, only the orthologs between *H. sapiens *and five common model organisms are presented (Table 1). These orthologs were based on the HomoloGene database. Interologs were determined from the model organisms *M. musculus *(mouse), *R. norvegicus *(rat), *D. melanogaster *(fruit fly), *C. elegans *(worm) and *S. cerevisiae *(yeast).

**Table 1:** Numbers of ortholog shared by human and five model organisms

Species (Taxonomy ID)^a^	Number of Genes with Orthologs	Number of Shared Orthologs Groups^b^					
		
		*H. sapiens*	*M. musculus*	*R. norvegicus*	*D. melanogaster*	*C. elegans*	*S. cerevisiae*
*H. sapiens *(9606)	19 491	**19 491 (100%)**	**16 330 (83.78%)**	**15 116 (77.55%)**	5039 (25.82%)	3951 (20.27%)	1593 (8.17%)
*M. musculus *(10090)	19 142	**16 330 (85.31%)**	**19 142 (100%)**	**16 674 (87.11%)**	4990 (26.07%)	3942 (20.59%)	1607 (8.39%)
*R. norvegicus *(10116)	17 766	**15 116 (85.08%)**	**16 674 (93.85%)**	**17 766 (100%)**	4662 (26.24%)	3711 (20.89%)	1509 (8.49%)
*D. melanogaster *(7227)	7794	5039 (64.65%)	4990 (64.02%)	4662 (59.82%)	**7794 (100%)**	**3377 (43.33%)**	1344 (17.24%)
*C. elegans *(6239)	4971	3951 (79.48%)	3942 (79.30%)	3711 (74.65)	**3377 (67.93%)**	**4971 (100%)**	1189 (23.92%)
*S. cerevisiae *(4932)	4589	1593 (34.71%)	1607 (35.01%)	1509 (32.88%)	1344 (29.29%)	1189 (25.91%)	**4589 (100%)**

^a^These species are ranked by the number of genes with ortholog information available.

^b^The percentage below the number of ortholog refers to the coverage on the species given in the left most column.

Based on ortholog information, the conservation of genes and ortholog groups among 18 eukaryotic species were identified. We found 81 genes that were conserved in all 18 species presented in HomoloGene (Additional File 2: Table S2), suggesting that these genes are fundamental and/or vital to eukaryotes. Interestingly, 243 genes are missing in *P. falciparum*, but found in the other 17 species, including members of the proteosome, various ATP synthases and many mitochondria-related genes. While most species in the HomoloGene database share a high proportion of orthologs with other species (ranging from 48.3% in *O. sativa *to 87.4% in *H. sapiens*), less than 20% of the 5,266 genes in *P. falciparum *can be grouped with genes from other species. This suggests that the lifestyle and biological processes of this parasite deviate from those of other organisms.

### PPIs in the POINT database

PPIs from the various model organisms were used to infer PPIs (interologs) in higher order organisms such as *H. sapiens*. Because experimental PPIs from the target organisms are needed to verify these inferred PPIs, collections of PPIs are essential for an ortholog-based approach. The POINT database has collected most of the available public PPI data for a range of organisms (Table 2). It contains more than 44,000 human PPIs with available ortholog information. In addition, more than 70,000 yeast interactions are available, suggesting that a considerable number of human interologs can be inferred. Most of these interactions were obtained from high-throughput techniques such as yeast two-hybrid screening, which is prone to a high rate of false positives and other errors. Within the high-confidence dataset, where only PPIs verified by two or more methods or reported in the literature two or more times are included, there are 28,559 human PPIs and 25,612 yeast PPIs with available ortholog information.

**Table 2:** Protein-protein interactions collected in the POINT database.

Species (Taxonomy ID)^a^	All Available PPIs		Confident PPIs	
		
	PPI	Orthologs Groups PPI^b^	PPI	Orthologs Groups PPI^b^
*S. cerevisiae *(4932)	82 445	70 264	31 162	25 612
*H. sapiens *(9606)	45 378	44 251	29 074	28 559
*D. melanogaster *(7227)	29 342	14 071	1106	764
*C. elegans *(6239)	5267	1572	692	288
*M. musculus *(10090)	3851	3746	1320	1291
*P. falciparum *(36329)	2844	188	8	8
*R. norvegicus *(10116)	1469	1399	1003	964
*A. thaliana *(3702)	1420	691	353	223
*S. pombe *(284812)	356	227	163	98
*G. gallus *(9031)	43	41	17	16
*O. sativa *(39947)	49	33	1	1
*C. familiaris *(9615)	2	2	1	1

^a ^These species are ranked by the number of available PPIs, except for Others and Inter-species.

^b ^Orthologous Group PPIs are PPIs with ortholog information available.

While the use of high-confidence PPIs eliminates many potential PPIs that are present in the available datasets, this trimming process reduces the false positive rate. Among the organisms listed in Table 2, *S. cerevisiae *shows the most dramatic drop in the number of PPIs when only high-confidence PPIs are selected. The reason for this is obvious: this species is a single cell organism. Most of the PPI datasets were obtained using high-throughput approaches, and have not been verified by other methods or reported independently in the literature. For *H. sapiens*, the number of high-confidence PPIs exceeds even those of yeast. However, some species in the HomoloGene database do not have PPI data available. For example, *P. troglodytes *(Chimpanzee) and *C. familiaris *(dog) have no inferred human interologs despite the large number of orthologs they share with *H. sapiens*.

### Interologs inferred from ortholog pairs

Given n objects in an undirected network (graph), there will be n(n-1)/2 relationships among these n objects and n*n relationships for a directed network. Since there are 19,491 human ortholog groups (Table 1), we therefore can assume that there are 19,491*(19,491-1)/2 pairwise interactions among these gene products. Certainly, a complete graph is not reasonable or biologically feasible. However, we can assume that each interaction can be associated with a probability and that the probability for a non-interacting pair will be 0. At this stage, we do not have enough information to assign a probability for each theoretical interaction. However, we can expand all 189,939,795 interactions among these 19,491 orthologous groups.

The interologs were inferred from ortholog information. Using the orthologous groups shared by humans and other species, we can obtain the maximum number of interologs from currently available interactomes. Only two orthologous groups shared by more than two species can be used to infer interologs. For example, if orthologous group A is shared by humans and mice, and orthologous group B is also shared by humans and mice, there will be a potential interolog A-B between humans and mice, although the probabilities associated with these two interactions (one in human and one in mouse) are not known.

Based on this assumption, we analyzed a number of orthologous group pairs and identified a number of species sharing these orthologous groups for *H. sapiens *(Additional File 3: Table S3). Among the 189,939,795 interactions, 180,191,177 interologs were inferred from ortholog information. This translates to 94.86% coverage of interologs (*IC*^*HSA*^). Although the theoretical interolog coverage is high (nearly 95%), the interolog coverage on currently available PPIs is not significant. For all available human PPIs, only 3,859/44,251 interactions (8.72%) can be inferred from known interactions in other model organisms. Using the trimmed set of high-confidence PPIs, this coverage drops to 4.61% (1,316/28,559). There is an obvious gap between the theoretical upper boundary and the experimentally observed data.

To investigate the origin of this gap, we further analyzed the interolog coverage of each model organism. Five common model organisms were selected. The number of inferable interologs, experimental PPI derived interologs and their interolog coverage were calculated (Table 3 and Table S3). It is interesting that the most commonly used model organism, *S. cerevisiae *(yeast), has a theoretical interolog coverage of only 0.67% (interologs inferred from yeast divided by all human interactions), whereas the *IC*^*HSA *^of *M. musculus *(mouse) and *R. norvegicus *(rat) are larger by two-orders of magnitude. However, for experimental human PPIs, the *IC*^*HSA *^of mouse is only 2-fold higher than that of yeast, and the *IC*^*HSA *^of rat is lower than that of yeast. The species contributions, *C*^*Sp*^, shown in this table are also informative. While mouse contributes 43.07% of the known interologs, yeast contributes only 19.85%. This trend was mostly unchanged for high-confidence PPIs, except the contribution of yeast was boosted to 32.29%.

The mapping of all orthologous group pairs permits interolog prediction for various eukaryotic species. For example, in the POINeT web service , interologs can be inferred for seven eukaryotic species (*H. sapiens*, *M. musculus*, *D. melangaster*, *C. elegans*, *S. cerevisiae*, *A. thaliana*, and *P. falciparum*). Currently, more than 32,000 human interologs can be inferred. Among them, 3,859 have been confirmed by experimental evidence. The continual growth of interactomes in every eukaryotic species will continue to improve the ability to predict interologs.

**Table 3:** Contributions of model organisms to human theoretical and experimental interologs.

Species (Taxonomy ID)	Theoretical Interologs Coverage^a^			Experimental PPIs and Interologs^b^					
		
	HomoloGene	OrthoMCL	TIGR EGO	All Available PPIs			Confident PPIs		
	*IC*^*HSA*^	*IC*^*HSA*^	*IC*^*HSA*^	Interologs	*IC*^*HSA*^	*C*^*SP*^	Interologs	*IC*^*HSA*^	*C*^*SP*^
*H. sapiens *(9606)	94.86%	100.00%	95.78%	3859	8.72%	N/A	1316	4.61%	N/A
*M. musculus *(10090)	70.19%	77.56%	50.24%	1662	3.76%	43.07%	551	1.93%	41.86%
*R. norvegicus *(10116)	60.14%	71.59%	35.83%	480	1.08%	12.44%	251	0.88%	19.07%
*D. melanogaster *(7227)	6.68%	12.01%	4.79%	766	1.73%	19.85%	92	0.32%	7.00%
*C. elegans *(6239)	4.11%	8.05%	2.78%	231	0.52%	5.99%	29	0.10%	2.20%
*S. cerevisiae *(4932)	0.67%	2.02%	0.77%	766	1.73%	19.85%	425	1.49%	32.29%

^a^IC^HSA ^for theoretical interologs are the number of interologs divided by all theoretical human PPIs derived from each ortholog databases.

^b ^IC^HSA ^for all available and confident experimental interologs are the number of interologs divided by available and confident human PPIs.

### Prediction of inter-species host-pathogen interactions

*P. falciparum *is a parasite with a complex life cycle, and this malarial parasite threatens millions of lives worldwide. Based on the HomoloGene database, *P. falciparum *has the least similar genome in comparison to other species. Only roughly 20% (990/5,266) of the genes share orthologous groups with other organisms. This suggests that many cellular processes vital to other eukaryotes may be missing or replaced in *P. falciparum*, and the interplay between the parasite and its two hosts may compensate for the functions missing in the *P. falciparum *genome. The interactome of P. falciparum has been determined experimentally [18] and modeled genome-wide [19]. This allows comparisons to be done between the genomes and interactomes of *P. falciparum *and its two hosts, *H. sapiens *and *A. gambiae *(the African malaria mosquito).

**Figure 1:**
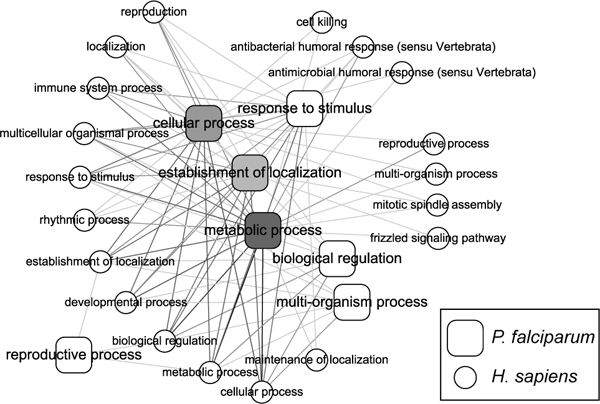
**Interactions between *P. falciparum *and *H. sapiens *are grouped by biological processes from Gene Ontology**. Interactions between *P. falciparum *and *H. sapiens *are grouped by biological processes from Gene Ontology. Each node represents a GO biological process in either *P. falciparum *or *H. sapiens*. The nodes of biological processes for *P. falciparum *are shaded based on their involvement in the inter-species interaction network; darker color implies larger involvement. For *P. falciparum*, most of the interactions are related to metabolic and cellular processes.

Using the experimental PPIs and interologs, 3,090 inter-species interactions between *P. falciparum *and *H. sapiens *(and not intra-*P. falciparum *interactions) were found (Additional File 4: Table S4). The Gene Ontology annotations of the *P. falciparum *and *H. sapiens *genes were identified. These inter-species PPIs have been grouped based on the ontology of their biological processes. The resulting network is illustrated in Figure 1. The nodes in Figure 1 are biological processes from *P. falciparum *and *H. sapiens*. Links between *P. falciparum *and *H. sapiens *biological processes were derived from interactions linking two genes that participate in the respective biological processes in the two species. Darker lines indicate the involvement of more interactions, allowing more interplay between the two biological processes. The *P. falciparum *biological processes are shaded using different levels of grey. Darker nodes indicate that more genes are involved in the process. In Figure 1, the metabolic processes and cellular processes of *P. falciparum *are most abundant in the host-parasite interaction network. This is understandable, since *P. falciparum *is a parasite and needs to acquire nutrients from the host erythrocyte. In the genomic-wide model of the *P. falciparum *interactome, only a small fraction of intra-*P. falciparum *interactions contributed to metabolic processes [19], which supports the notion that *P. falciparum *metabolic processeses may be dependant on human metabolic and cellular processes. There are also other interesting interactions between *P. falciparum *and the antimicrobial, antibacterial, cell killing and immune system processes of *H. sapiens*.

### Filtering and analysis of predicted inter-species interactions

**Figure 2:**
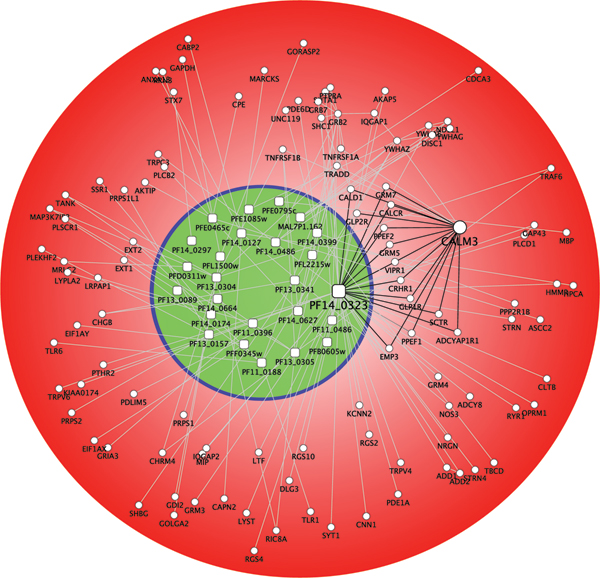
**Illustration of filtered *H. sapiens*-*P. falciparum *interactions**. *P. falciparum *calmodulin (PF14_0323) shares 13 interaction partners with human calmodulin (CALM3), suggesting competition between the two proteins, and interference of host cell Ca^2+ ^homeostasis. (Red: red blood cell; Green: the parasitophorous vacuole).

Although more than 3,000 *H. sapiens*-*P. falciparum *PPIs were inferred, not all of these interactions are likely to take place under physiological conditions due to spatiotemporal constraints. Filtering using gene ontology annotations resulted in 918 host-pathogen interactions. Further filtering of *P. falciparum *sequences using the presence/absence of translocational signals led to 95 PPIs (Figure 2). Only 15 *P. falciparum *proteins participate in these 95 PPIs (Table 4). One of the *P. falciparum *proteins, calmodulin (PF14_0323), interacts with 50 human proteins. It is well known that *P. falciparum *requires an environment with high Ca^2+ ^levels [20], and the abundence of calmodulin-based interactions may help *P. falciparum *maintain this high Ca^2+ ^concentration [21]. Among the 50 human proteins interacting with PF14_0323, 13 also interact with human calmodulin (CALM3). This suggests that *P. falciparum *calmoduin shares some of the targets of human calmodulin, and may hijack these PPIs for its own purpose. The protein with the second highest number of interactions was N-myristoyltransferase (PF14_0127). Many proteins interacting with calmodulin require myristoylation in N-terminal [22-24], further supports the functioning of the calmodulin-centric network.

**Table 4:** *P. falciparum *proteins participate in 95 PPIs filtered from 918 host-pathogen interactions.

**Gene Symbol**	**Gene ID**	**Description**	**Number of Interactions**
PF14_0323	811905	Calmodulin	50
PF14_0127	811708	N-myristoyltransferase	6
PF14_0627	812209	ribosomal protein S3, putative	6
PF11_0188	810735	heat shock protein 90	6
PF14_0399	811981	ADP-ribosylation-like factor, putative	4
PF13_0157	814127	ribose-phosphate pyrophosphokinase	4
MAL7P1.162	2654986	dynein heavy chain, putative	4
PF14_0486	812068	elongation factor 2	3
PF11_0486	811029	MAEBL	3
PFF0345w	3885886	translation initiation factor IF-2, putative	2
PFE0795c	812973	nif-like protein, putative	2
PF11_0396	810942	Protein phosphatase 2C	2
PFB0605w	812721	Ser/Thr protein kinase, putative	1
PF14_0664	812246	biotin carboxylase subunit of acetyl CoA carboxylase, putative	1
PF14_0297	811879	ecto-nucleoside triphosphate diphosphohydrolase 1, putative	1

Previously, Dyer *et al. *[16] have inferred host-pathogen interactions using Bayesian statistics. *H. sapiens*-*P. falciparum *PPIs predicted by the Bayesian approach are mainly enriched in 'blood coagulation' and 'membrane integration' protein interactions. This may partly be due to the gene ontology terms used to filter the PPIs. It is difficult to compare the two works, since the datasets and methodology used are different. However, the intersection of the two datasets reveals 3 interactions between PF14_0359 and the TNF receptor associated factor family (TRAF1, TRAF2 and TRAF6). PF14_0359 is a hypothetical protein. Inspection of the HomoloGene database reveals that PF14_0359 may be a homolog of DNAJA1 (HSP40). The functional implications of these three interactions require further investigation. However, TNF associated factor family are known to be involved in host immune response, suggesting that *P. falciparum *may interfere with this defence mechanism in *H. sapiens*. All in all, the diversity of different host-pathogen interaction inference methods suggests that these and other approaches may complement each other. And further development of the ability to predict host-pathogen interactions may benefit from the combination of multiple diverse approaches.

## Conclusion

The expansion of all orthologous pairs enables the inference of interologs for various eukaryotic organisms, as illustrated by POINeT . The same inference method can also be applied to the prediction of inter-species interaction, especially in the case of host-pathogen interactions. The *H. sapiens*-*P. falciparum *PPIs inferred in our work reveal that *P. falciparum *may utilize calcium modulating proteins in the host cell to maintain Ca^2+ ^levels, and this may serve as a target for drug development strategies [25].

## Methods

### Ortholog information for interolog analysis

One of the limitations inherent in the analysis of interologs is the assignment of the orthologs, which is achieved using various BLAST algorithms together with several additional criteria [6,9,11,26,27] or from the NCBI HomoloGene and other protein/gene cluster databases. In this work, the ortholog information for each human gene was identified using the NCBI HomoloGene Release 54 [28]. The NCBI HomoloGene database contains homologous information for 18 eukaryotic organisms and has been augmented with homology and phenotype information drawn from various sources, e.g., MGI [29] and Fly base [30].

### Collection of PPIs

The new version of POINT integrated several publicly accessible PPI datasets (Additional File 5: Table S5). These data sources have diverse entry formats, disparate ID systems and different protein symbols. The diversity of these datasets made the task of performing cross-site browsing or iterative querying very tedious and challenging. We systematically re-organized these datasets to improve and standardize the publicly accessible PPIs. High-throughput PPI datasets are prone to false positives and errors. Therefore, we also generate a relatively high-confidence PPI subset, which refers to a PPI subset where the PPIs have been verified by two or more experimental methods or published in the literature two or more times.

### Evaluation of interolog coverage

The interolog coverage is quantifiable from an estimation of the ortholog-based PPI prediction power. The definition of interolog coverage is as follows:

$$IC^{HSA}=\frac{N}{T^{HSA}}\times 100\%$$

where *T*^*HSA *^is the total number of human (*H. sapiens*) interactions (whether theoretical, experimental, or highly confident), *N *is the number of interologs, and *IC*^*HSA *^is the interolog coverage for the human interactome. Another measure is the contribution of a given model organism to the human interologs and this is defined as

$$C^{Sp}=\frac{I^{Sp}}{TI^{HSA}}\times 100\%$$

where *TI*^*HSA *^is the total number of human interologs, *I*^*Sp *^is the number of interologs inferred from species *Sp*, and *C*^*Sp *^is the contribution of species *Sp *to human interologs.

### Inference and filtering of inter-species interactions

With the expanded orthologous pairs, intra- and inter-species PPIs can be inferred with ease. The inference of *H. sapiens*-*P. falciparum *interactions are based on orthologous pairs with one-side orthology to *P. falciparum*. For example, given a PPI between M_a _and M_b _in species M, if M_a _has an ortholog in *P. falciparum *(P_a_), and M_b _has an ortholog H_b _in *H. sapiens *(but not in *P. falciparum*), an interaction between P_a _and H_b _is inferred.

However, interologs inferred from orthologous pairs may not occur in vivo, especially in the case of inter-species interactions. *P. falciparum *inhabits a parasitophorous vacuole after its entry into the red blood cell. A translocational signal peptide (RELXE/Q) is required to translocate *P. falciparum *proteins into red blood cell cytoplasm for host-cell manipulation [31-33]. Also, proteins localized in the nucleus (both *H. sapiens *and *P. falciparum*) are not likely to participate in inter-species PPIs. Two filters have been applied to reduce such unlikely cases. The first filter utilizes gene ontology annotations. Human proteins with the following annotations were removed: mitochondria, nucleus, ribosome, cell process, helicase activity, complex, nuclease activity, nucleic acid binding, nucleotide binding or proteolysis. The second filter utilizes the translocation signal RELXE/Q, where X refers to any amino acids. *P. falciparum *sequences matching this pattern within the first 25% of its length are kept, since translocation signals are likely to appear at the N-terminal.

## Competing interests

The authors declare that they have no competing interests.

## Authors' contributions

CYH, CYK, FSW, and JML provide the concept and guidelines for the POINT/POINeT web servers. SAL collects and analyzes the protein-protein interaction and ortholog data, and predicts the inter-species interaction data. CHC proposes the inter-species and host-pathogen concept and writes the manuscript. CHT provides the literature about *P. falciparum*.

## Supplementary Material

Additional file 1 Table S1. The orthologous group coverage among 18 eukaryotic species. Click here for file

Additional file 2 Table S2. Orthologous groups conserved in multiple species. Click here for file

Additional file 3 Table S3. Interlog coverage and contributions for each species. Click here for file

Additional file 4 Table S4. Predicted inter-species interactions between *P. falciparum *and *H. sapiens*. Click here for file

Additional file 5 Table S5. Protein-protein interaction data sources. Click here for file
